# Transcriptomic Analyses Reveal Insights into the Shared Regulatory Network of Phenolic Compounds and Steviol Glycosides in *Stevia rebaudiana*

**DOI:** 10.3390/ijms25042136

**Published:** 2024-02-10

**Authors:** Samuel Simoni, Alberto Vangelisti, Clarissa Clemente, Gabriele Usai, Marco Santin, Maria Ventimiglia, Flavia Mascagni, Lucia Natali, Luciana G. Angelini, Andrea Cavallini, Silvia Tavarini, Tommaso Giordani

**Affiliations:** 1 Department of Agriculture, Food and Environment (DAFE), University of Pisa, Via del Borghetto, 80, 56124 Pisa, Italyclarissa.clemente@phd.unipi.it (C.C.); marco.santin@unipi.it (M.S.); maria.ventimiglia@phd.unipi.it (M.V.); silvia.tavarini@unipi.it (S.T.); 2 Interdepartmental Research Centre “Nutraceuticals and Food for Health—NUTRAFOOD”, University of Pisa, Via del Borghetto, 80, 56124 Pisa, Italy

**Keywords:** stevia rebaudiana, secondary metabolites, steviol glycosides, phenolic compounds, RNA-seq analysis

## Abstract

*Stevia rebaudiana* (Bertoni) is a highly valuable crop for the steviol glycoside content in its leaves, which are no-calorie sweeteners hundreds of times more potent than sucrose. The presence of health-promoting phenolic compounds, particularly flavonoids, in the leaf of *S. rebaudiana* adds further nutritional value to this crop. Although all these secondary metabolites are highly desirable in *S. rebaudiana* leaves, the genes regulating the biosynthesis of phenolic compounds and the shared gene network between the regulation of biosynthesis of steviol glycosides and phenolic compounds still need to be investigated in this species. To identify putative candidate genes involved in the synergistic regulation of steviol glycosides and phenolic compounds, four genotypes with different contents of these compounds were selected for a pairwise comparison RNA-seq analysis, yielding 1136 differentially expressed genes. Genes that highly correlate with both steviol glycosides and phenolic compound accumulation in the four genotypes of *S. rebaudiana* were identified using the weighted gene co-expression network analysis. The presence of UDP-glycosyltransferases *76G1*, *76H1*, *85C1*, and *91A1*, and several genes associated with the phenylpropanoid pathway, including *peroxidase*, *caffeoyl-CoA O-methyltransferase*, and *malonyl-coenzyme A:anthocyanin 3-O-glucoside-6″-O-malonyltransferase*, along with 21 transcription factors like *SCL3*, *WRK11*, and *MYB111*, implied an extensive and synergistic regulatory network involved in enhancing the production of such compounds in *S. rebaudiana* leaves. In conclusion, this work identified a variety of putative candidate genes involved in the biosynthesis and regulation of particular steviol glycosides and phenolic compounds that will be useful in gene editing strategies for increasing and steering the production of such compounds in *S. rebaudiana* as well as in other species.

## 1. Introduction

*Stevia rebaudiana* (Bertoni) is a perennial shrub of the Asteraceae family distributed across Asia, Europe, and South and North America. It is a valuable crop, well known for accumulating, in its leaves, steviol glycosides (SGs), up to 20% of the total dry weight [1], which are no-calorie sweeteners hundreds of times more potent than sucrose, of significant commercial value. It is also known for the significant amount of beneficial health-related phenolic compounds (PCs) in its leaves [2]. *S. rebaudiana* can be an annual or perennial herbaceous plant, depending on the environmental conditions [3]. It is an allogamous species with entomophilous pollination and exhibits flowers characterized by sporophytic self-incompatibility. Some cultivars cannot produce viable seeds; thus, vegetative propagation is the only way of reproduction, mainly from cuttings or micro-propagation [4]. Many countries, including Canada, Brazil, India, and Italy, have recently made attempts to cultivate *S. rebaudiana* [5]. China, South Asia, and South America are currently the main *S. rebaudiana*-producing areas in terms of both purified SGs and dry leaves as raw material. According to market watch, *S. rebaudiana* had a commercial value of USD 447 million in 2016 and was expected to reach USD 1045 million in 2023 (Southey, 2019: https://www.foodnavigator.com/Article/2019/02/14/Sustainable-stevia-Tate-Lyle-to-assess-eco-impact-of-sweetener; accessed on 17 March 2023).

In this context, SGs, serving as an alternative natural sweetener, can provide substantial benefits by preventing severe illnesses associated with modern lifestyles, such as diabetes, obesity, heart diseases, and hypertension [6,7,8]. To date, 64 SGs have been identified in different concentrations in extracts of *S. rebaudiana* leaves [9] (EFSA Panel on Food Additives and Flavourings (FAF), 2020), with stevioside and rebaudioside A being the most abundant, followed by rebaudioside B–E, dulcoside A, and steviolbioside. Since stevioside and rebaudioside A are abundant and a hundred times sweeter than sucrose, they are the two widely used SGs in the marketplace [10]. However, due to the liquorice aftertaste of stevioside, numerous attempts have been made to characterize more appealing rebaudiosides, besides rebaudioside A [11].

The early stages of SG biosynthesis have been clarified [12]. They comprise seven genes encoding proteins of the methyl erythritol-4-phosphate (MEP) pathway: deoxyxyulose-5-phosphate synthase, deoxyxyulose-5-phosphate reductoisomerase, 4-diphosphocytidyl-2-C-methyl-D-erythritol synthase, 4-diphosphocytidyl-2-C-methyl-D-erythritol kinase, 4-diphosphocytidyl-2-C-methyl-D-erythritol 2,4-cyclodiphosphate synthase, 1-hydroxy-2-methyl2(E)-butenyl 4-diphosphate synthase, and 1-hydroxy-2-methyl-2(E)-butenyl 4-diphosphate reductase. In the subsequent reactions, four proteins concur to the formation of kaurenoic acid: geranylgeranyl diphosphate synthase, ent-copalyl diphosphate synthase, kaurene synthase, and kaurene oxidase [1,13]. The SG biosynthetic pathway branches from the gibberellin biosynthesis, producing steviol, the precursor of the SGs, through the action of kaurenoic acid 13-hydroxylase [13]. At later stages of biosynthesis, several UDP-glycosyltransferases (UGTs) are involved in the glycosylation of steviol, resulting in the production of a plethora of SGs [13]. The five UGTs identified and clarified as being involved in the glycosylation of SGs are UGT74G1, UGT76G1, UGT73E1, UGT85C2, and UGT91D2 [11,14,15,16].

Similarly to SGs, PCs have drawn much attention due to their numerous beneficial biological functions, including their ability to scavenge free radicals, metal chelation, inhibition of cellular proliferation, modification of signal transduction pathways, and enzymatic activity [17]. Their regular consumption within the diet has been linked to a lower risk of different types of cancers, cardiovascular and neurodegenerative diseases, and several age-related disorders [18,19]. A *S. rebaudiana* leaf extract contains a significant amount of total PCs and shows extensive antioxidant activity [2,20,21,22]. More than 30 PCs found in *S. rebaudiana* leaves have been identified; the primary phenolic classes are flavonoids, chlorogenic acids, and a polyphenol family of esters that includes hydroxycinnamic acids and quinic acid [2,23,24,25,26,27]. In particular, flavonoids represent one of the most widespread groups of plant phenolics and serve as plants’ primary active nutraceutical ingredients. They exhibit strong antioxidant properties and metal chelation abilities. Additionally, they have been acknowledged for their anti-inflammatory, antiallergic, hepatoprotective, antithrombotic, antiviral, and anticarcinogenic activities [28].

The phenylpropanoid biosynthetic pathway results in the accumulation of various phenolic compounds. It has been constantly reconstructed in recent years [29,30,31]. As the first step of the biosynthesis of the phenylpropanoids, the phenylalanine ammonia-lyase deaminates phenylalanine into cinnamic acid. The subsequent reaction catalysed by cinnamate 4-hydroxylase produces *p*-coumaric acid. Then, the 4-coumarate-coenzyme A ligase links a coenzyme A to *p*-coumaric acid to generate *p*-coumaroyl-CoA [32,33]. This step represents a branch point to generate various classes of compounds, such as flavonoids and lignin. Chalcone synthase is the first enzyme to initiate the flavonoid biosynthesis. Other flavonoid key enzymes are represented by chalcone isomerase, flavanone 3-hydroxylase, dihydroflavonol 4-reductase, leucoanthocyanidin reductase, anthocyanidin synthase, and anthocyanidin reductase. On the other hand, lignin biosynthesis is carried out by several enzymes, including hydroxycinnamoyl transferase, caffeate/5-hydroxyferulate 3-O-methyltransferase, caffeoyl-CoA 3-O-methyltransferase, cinnamyl alcohol dehydrogenase, cinnamoyl-CoA reductase. These sequential activities convert the hydroxycinnamic acids into their respective hydroxycinnamic alcohols (or monolignols): the sinapyl, the coniferyl, and the *p*-coumaryl alcohols. These alcohols polymerize to form the complex lignin structure. Lastly, UGTs are able to glycosylate several PCs, such as flavonoids, monolignols, and catechin derivatives [34,35]. For example, UGT72E1, UGT72E2, and UGT72E3 were reported to be glucosyltransferases acting on coniferyl and sinapyl alcohols to form coniferyl alcohol-4-O-glucosides and sinapyl alcohol-4-O-glucosides, respectively. UGT83A1 catalyses the glucosylation of coniferyl alcohol, sinapyl alcohol, and *p*-coumaryl alcohol in rice, which is encoded by GSA1 [36].

Several studies on the expression of some specific genes have been conducted to investigate SG biosynthesis in *S. rebaudiana*, for example, real-time PCR and expressed sequence tag (EST) sequencing [14,37,38,39]. However, the RNA-seq technology is apparently the most effective in investigating gene expression at the -omic level [40,41]. In fact, contrary to other techniques, RNA-seq analyses provide a faster, cheaper, and reliable comprehensive report on the transcriptome. Although this approach allowed the identification of genes that are responsible for SG biosynthesis [11,15], the understanding of genes involved in PC biosynthesis and regulation is still limited in *S. rebaudiana*. Furthermore, the intricate shared regulatory network between these metabolic pathways still needs to be investigated.

Therefore, this study aimed to identify the genes encoding enzymes and regulatory molecules, including transcription factors (TFs), involved in PC biosynthesis and regulation, especially those that act synergistically with genes involved in SG biosynthesis. With this aim, a comparative RNA-seq analysis between four genotypes with different contents of these metabolites was carried out. In particular, three genotypes with a high overall content and one with a low overall content of PCs and SGs were analysed. Such candidate genes would be the targets for gene editing strategies aimed to increase both PC and SG biosynthesis or to change the relative proportions of these specialized metabolites in *S. rebaudiana* in order to increase the nutritional value of its extracts.

## 2. Results

### 2.1. Phytochemical Evaluation and Antioxidant Activity

The different phytochemical compositions among the four cultivars represented by the content of total SGs, total phenols, total flavonoids, and antiradical activity at the pre-flowering stage were measured (Table 1). The assessment of the antiradical activity relies on the capacity of antioxidant molecules to reduce the 1,1-diphenyl-2-picryl-hydrazil (DPPH) radical.

Among these genotypes, SL, L34, and PL exhibited a significantly higher total SG content than RG, which showed the lowest content of these metabolites. Regarding total phenols and DPPH activity, L34 showed the highest content together with SL, while RG exhibited the lowest content. Moreover, L34 revealed a higher content of total flavonoids, whereas RG had the lowest content of these metabolites among the four genotypes. Overall, SL, L34, and PL showed significantly higher SGs, total phenol content, and DPPH activity than RG (Table 1).

**Figure 1:**
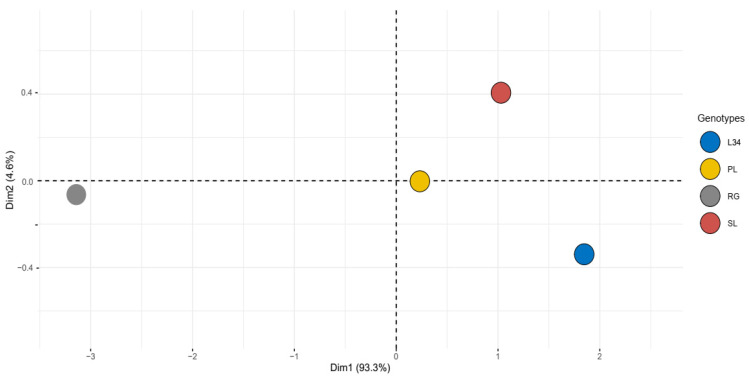
Principal component analysis (PCA) on total SGs, total phenols, total flavonoids, and DPPH activity of four *S. rebaudiana* genotypes.

In order to elucidate the patterns of variation among the four genotypes, a PCA analysis was performed for total phenols, flavonoids, steviol glycosides, and antiradical activity. The score plot obtained was defined by a PC1 and a PC2, covering 93.3% and 4.6% of the variance, respectively, for a total explained variance of 97.9% (Figure 1). The genotypes L34, SL, and PL belong to the same clustering pattern, clearly distinguished from RG, indicating a more pronounced difference in terms of their bioactive compound content.

An HPLC/UV-Vis analysis was conducted by comparing the retention times with authentic rubusoside, dulcoside A, stevioside, and rebaudioside A, C, D, and E standards to assess the different SG profiles of the four *S. rebaudiana* genotypes (see Section 4). The results are shown in Table 2.

Stevioside and Reb A were confirmed to be abundant in the *S. rebaudiana* leaf, significantly affecting the total content of SGs. RG showed the lowest amount of these two metabolites whereas L34 presented the highest content. SL, followed by PL and L34, were the genotypes with the highest content of Reb A. Reb M was mainly found in traces. The content of Reb D was higher in PL. Notably, Reb E was not detected in RG, whereas it was present in L34, SL, and PL (Table 2).

### 2.2. Transcriptomic Analyses

#### 2.2.1. cDNA Sequencing and Mapping on *S. rebaudiana* Reference Transcriptome

The four genotypes were analysed concerning gene expression using Illumina RNA-seq. Illumina sequencing generated 12 libraries, yielding a total of 339,515,517 paired-end reads of 150 nt, with an average of 28,292,959 reads per library (Supplementary Table S1). After trimming, 218,736,166 reads 120 nt long were filtered, obtaining an average of 18,228,013 reads per library (Supplementary Table S1). This number of reads is suitable to perform comparative expression analyses [42]. After mapping, an average of 79% high-quality reads, cleaned of rRNA sequences, were aligned on coding sequences (CDSs) of *S. rebaudiana* [43].

#### 2.2.2. Analysis and GO Enrichment of Differentially Expressed Genes

We evaluated the expression of the 44,112 coding sequences identified in the assembled genome of *S. rebaudiana* [43]. The expressed genes totalled to 29,487 (sequences with RPKM > 1 in at least one library).

The comprehensive PCA of total SGs, total phenols, total flavonoids, and DPPH activity indicated a clear difference between RG and the other genotypes (PL, SL, and L34). Therefore, we studied differential gene expression by performing independent pairwise comparisons of PL, SL, and L34 to the RG genotype, used as a common reference. 

From these three comparisons, we initially retrieved a total pool of 1088 over-expressed (OE) genes and 992 under-expressed (UE) genes (Supplementary Figure S1). Any DEG specific to the comparisons between PL, SL, and L34, without taking into account RG, was then excluded from the common pool. This step allowed us to identify genes involved in SG and PC accumulation in the three genotypes, regardless of the specific genotype. The functional characterization was conducted on the resulting filtered shared pool of 1136 DEGs (Supplementary Table S2). In particular, the filtered shared pool comprised 606 OE and 530 UE genes.

The GO enrichment analysis revealed statistically significant differences in GO terms for OE and UE genes (Supplementary Figure S2). In particular, the most frequent enriched OE GO terms were ‘transferase activity’ (GO:0016740), ‘organonitrogen compound metabolic process’ (GO:1901564), and ‘transferase activity, transferring phosphorus-containing groups’ (GO:0016772). Conversely, the most abundant UE GO terms were ‘catalytic activity’ (GO:0003824), ‘transferase activity’ (GO:0016740), and ‘cation binding’ (GO:0043169) (Supplementary Figure S2).

#### 2.2.3. Functional Characterization of DEGs Related to Primary and Secondary Metabolism

**Figure 2:**
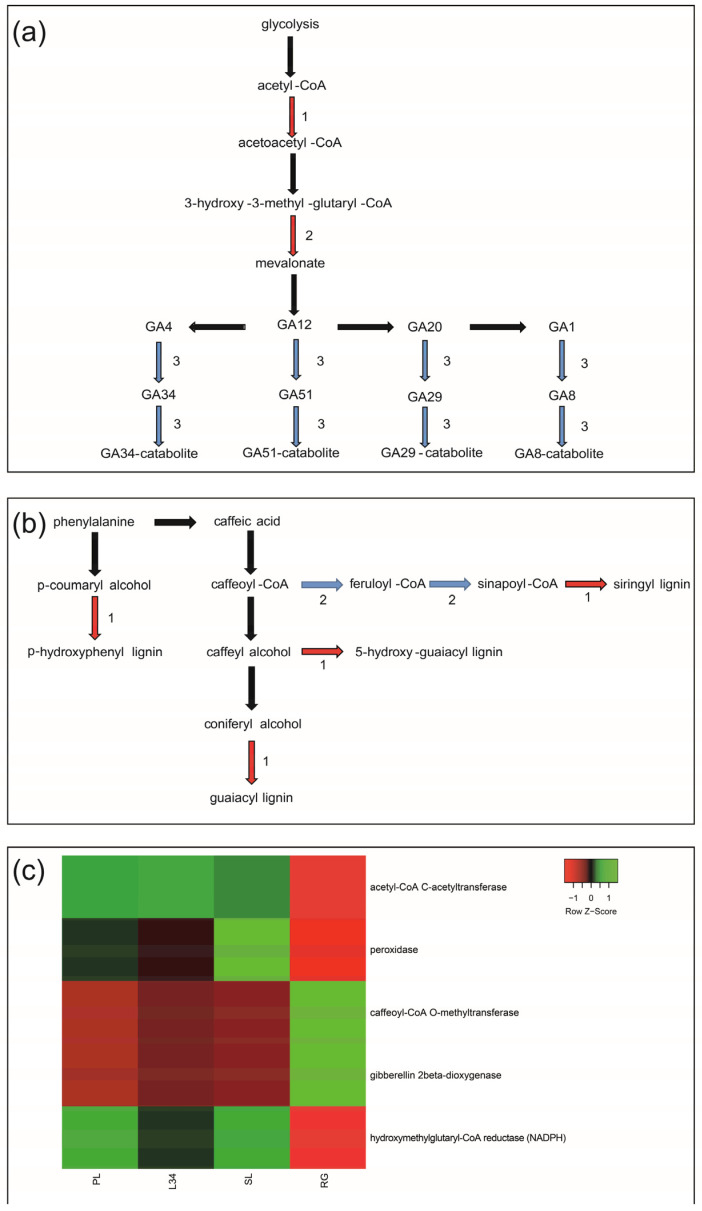
Schematisation of the KEGG pathways. The black arrows indicate the formation of products in the metabolic chain. The red arrows indicate the genes encoding enzymes that were over-expressed. The blue arrows indicate the genes encoding enzymes that were under-expressed. (**a**) ‘terpenoid and diterpenoid’ pathways; OE: (1) *acetyl-CoA C-acetyltransferase*; (2) *hydroxymethylglutaryl-CoA reductase (NADPH)*; UE: (3) *gibberellin 2-beta-dioxygenase*; (**b**) ‘phenylpropanoid’ pathway; OE: (1) *peroxidase*, UE: (2) *caffeoyl-CoA O-methyltransferase*; (**c**) heatmap representing the expression pattern of DEGs (RPKM) belonging to ‘terpenoid and diterpenoid’ and ‘phenylpropanoid’ pathways in the four genotypes.

We performed KEGG and MapMan analyses to compare the functional classes using the previously identified DEGs. The ‘terpenoid and diterpenoid’ KEGG pathways revealed two OE genes involved in the early stages of SG biosynthesis encoding *acetyl-CoA C-acetyltransferase* and *hydroxymethylglutaryl-CoA reductase* (*NADPH*) (Figure 2a). Furthermore, a UE gene encoding *gibberellin 2-beta-dioxygenase* (*GA2OX2*) was identified within the diterpenoid pathway (Figure 2a).

The KEGG pathway known as ‘phenylpropanoid’ was examined (Figure 2b). One OE gene encoding for peroxidase, involved in lignin production, and one UE caffeoyl-CoA O-methyltransferase were found to be involved in the phenylpropanoid pathway (Figure 2b). Figure 2c reports the heatmap representing the expression pattern of DEGs (RPKM) belonging to ‘terpenoid and diterpenoid’ and ‘phenylpropanoid’ pathways in the four genotypes.

**Figure 3:**
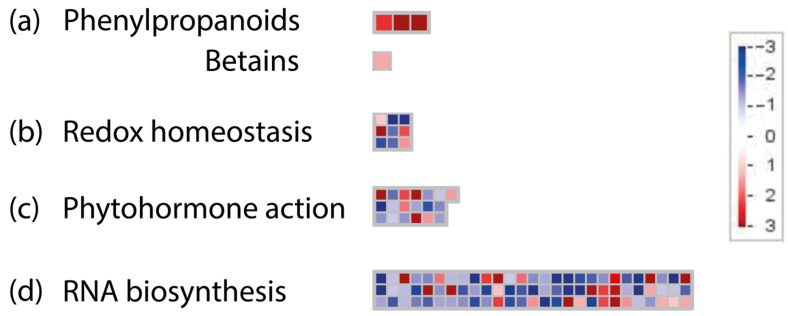
Analysis of DEGs using MapMan. (**a**) ‘Secondary metabolites’. (**b**) ‘Redox homeostasis’. (**c**) ‘Phytohormone action’. (**d**) ‘RNA biosynthesis’. The red squares represent over-expressed genes, while the blue squares represent under-expressed genes. The scale is based on the logarithmic fold change (FC) of the genes and ranges from dark blue (log_2_FC = −3) to dark red (log_2_FC = 3).

The MapMan analysis revealed four DEGs related to bin ‘Secondary metabolism’ (Figure 3). In particular, three OE genes belonging to ‘Phenylpropanoids’ were involved in pre-mRNA splicing (Figure 3a; Supplementary Table S3). The maps corresponding to ‘Redox homeostasis’ showed OE genes coding for antioxidant enzymes’ class with the capacity to scavenge free radicals, such as *glutathione peroxidase* (*GPX*) and *ascorbate peroxidase* (*APX*) (Figure 3b; Supplementary Table S3). Within ‘Phytohormone action’ (Figure 3c; Supplementary Table S3), the gene encoding for the abscisic acid receptor recruitment factor (CAR) was OE. On the contrary, one gene involved in the GA synthesis encoding *gibberellin 2-oxidase* (*GA2ox*), two transcriptional repressors of auxin synthesis, and one auxin signal mediator (*ARF*) were UE. The ‘RNA biosynthesis’ bin showed 76 transcription factors differentially expressed belonging to several families, such as WRKY, MYB, ERF, and bHLH (Figure 3d; Supplementary Table S3). The expression patterns of DEGs (RPKM) belonging to all analysed MapMan bins in the four genotypes are reported in Supplementary Figure S3.

#### 2.2.4. Analysis of Gene Networks and Correlation with Biochemical Traits

**Figure 4:**
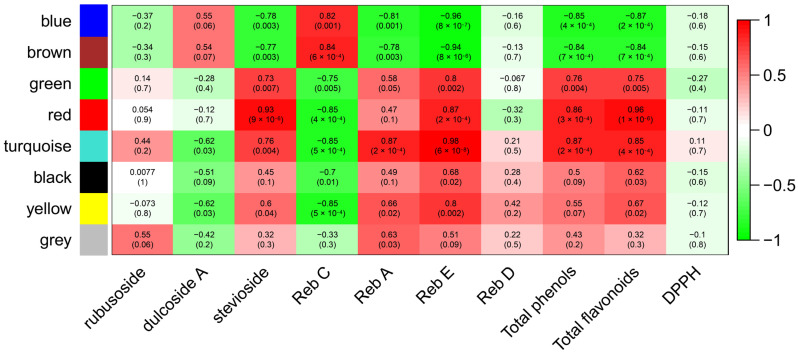
Association of gene modules to biochemical traits. Each row corresponds to a module named after an assigned colour, and each column corresponds to a specific biochemical trait. Each cell shows a correlation and *p*-value, in brackets, between the gene network and biochemical trait. The table is coded through correlation according to the colour legend, spanning from brighter red for the highest correlation to brighter green for the lowest correlation.

A weighted gene co-expression network analysis (WGCNA) was used to find co-expression modules significantly associated with the previously investigated biochemical traits. Overall, we identified eight modules (Supplementary Figure S4): black, blue, brown, green, grey, red, turquoise, and yellow, consisting of 52, 271, 135, 78, 6, 54, 449, and 91 eigengenes, respectively. The eigengene is the first principal component of all genes in a module and can be considered a representative expression profile for the genes in the module. In particular, the turquoise module (449 eigengenes) exhibited a high correlation with Reb A (r = 0.87, *p*-value = 2 × 10^−4^), Reb E (r = 0.98, *p*-value = 6 × 10^−8^), total phenols (r = 0.87, *p*-value = 2 × 10^−4^), and total flavonoids (r = 0.85, *p*-value = 4 × 10^−4^) (Figure 4). Within the turquoise module, we found two *UGT76H1*, one *UGT76G1*, and one *UGT85C1*, in addition to 21 TFs, and several genes belonging to the phenylpropanoid pathway, such as *peroxidase* (*PER17*), *caffeoyl-CoA O-methyltransferase* (*CAMT*), and the *malonyl-coenzyme A:anthocyanin 3-O-glucoside-6″-O-malonyltransferase* (*3MAT*).

**Figure 5:**
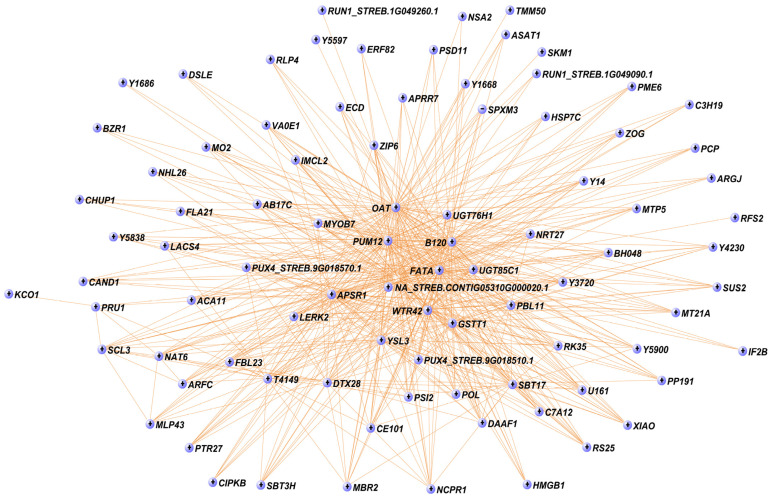
Co-expression network of the turquoise module eigengenes. The network shows the interaction that was detected between the eigengenes in the module with a cutoff of 0.4. Each node represents a gene in the network. NA: not annotated.

Interestingly, the core of the turquoise module (cutoff = 0.4) included 88 genes (Figure 5). Among them, we found two UGTs (*UGT85C1* and *UGT76H1*) and several TFs, including *ARFC*, *BH048*, *C3H19*, *DSLE*, *ERF82*, and *SCL3* (Figure 5). In addition to the UGTs, at the centre of the gene network, there is a gene not yet annotated (NA_STREB.CONTIG05310G000020.1) (Figure 5). On the other hand, the red module showed high correlation with stevioside (r = 0.93, *p*-value = 9 × 10^−6^), Reb E (r = 0.87, *p*-value = 2 × 10^−4^), total phenols (r = 0.86, *p*-value = 3 × 10^−4^), and total flavonoids (r = 0.96, *p*-value = 1 × 10^−6^) (Figure 4). The red module included the *shikimate O-hydroxycinnamoyltransferase* (*HST*), involved in the phenylpropanoid pathway, and the TF *WRKY6*, along with 13 unknown genes (NA) (Supplementary Figure S5).

#### 2.2.5. Characterization of Differentially Expressed UGTs

We deepened the DEGs’ functional characterization by identifying the differentially expressed UGTs, which have a central role in the biosynthesis of SGs. Specifically, we found four OE UGTs, UGT85C1, UGT83A1, UGT76H1, and UGT76G1 (Supplementary Table S2). In contrast, we detected six UE UGTs: UGT92A1, UGT91A1, UGT90A1, UGT83A1, UGT76H1, and UGT73E1 (Supplementary Table S2). UGT76H1 was identified as both OE and UE because they represent distinct transcripts originating from paralogous genes.

To infer the putative function of the 10 differentially expressed UGTs, we constructed a dendrogram based on their amino acid sequences, comparing them with UGTs known to be involved in SGs and phenylpropanoid metabolism collected from current literature.

**Figure 6:**
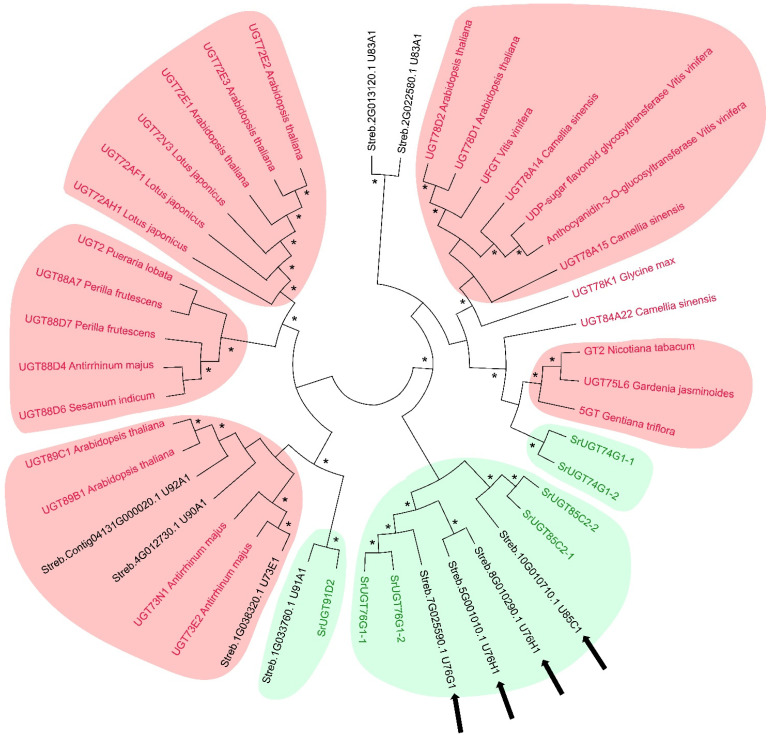
Dendrogram based on the amino acid sequences of differentially expressed UGTs (written with black characters) and several UGTs with known function in the metabolic pathways of SGs (UGTs written with the green characters) and phenylpropanoids (UGTs written with red characters) characterised in other studies. Black arrows indicate UGTs belonging to the turquoise module (see text). The clusters of UGTs with a role in phenylpropanoid biosynthesis are highlighted with a red background. The clusters of UGTs with a role in SG biosynthesis are highlighted with a green background. * indicates Bootstrap values above 60%.

The dendrogram revealed eight statistically significant clusters (>60% bootstrap), including three clusters consisting of UGTs involved in SG biosynthesis and five clusters of UGTs related to phenylpropanoid metabolism (Figure 6). Specifically, three DEGs encoding UGTs belong to the phenylpropanoid-glycosylation-related clusters. In the clusters of SG-production-related UGTs, five DEGs were identified. Noteworthily, UGT85C1 and two UGT76H1 (belonging to the turquoise module, see Section 2.2.4) fall in the cluster in which *S. rebaudiana* UGT85C2 and UGT76G1 are found, indicating a putative role in SG biosynthesis of these enzymes (Figure 6).

## 3. Discussion

### 3.1. Genes Putatively Involved in the Quantitative Variation of Steviol Glycosides and Phenolic Compounds in S. rebaudiana Leaf

Biochemical analyses on SG and PC content among four genotypes of *S. rebaudiana* showed profound differences in total SGs, total phenols, total flavonoid content, and antiradical activity. Genotype RG showed the lowest values of these parameters, whereas PL, SL, and L34 showed the highest ones (Table 1, Figure 1).

These differences reflect significant variations in stevioside and Reb A content, the most abundant SGs, but also Reb E, which was not detectable in RG. Elevated levels of total SGs, notably Reb A and Reb E, in SL, L34, and PL suggest enhanced organoleptic characteristics that are highly valued and beneficial for the agro-food processing industry. Furthermore, a higher content of total phenols, flavonoids, and antiradical activity entails a higher antioxidant and antiradical capacity of SL, L34, and PL leaves. The increase in these compounds in *S. rebaudiana* leaves enhances nutritional and medicinal applications thanks to their capacity to delay the oxidative degradation of lipids and to improve the shelf life of foods and beverages [44].

To identify candidate genes involved in the regulation of SG and PC content in *S. rebaudiana* leaves, based on observed biochemical differences, we performed transcriptomic analyses through independent pairwise comparisons between RG and PL, SL, and L34. These comparisons allowed us to determine core genes involved in differences in total SGs, phenols, and flavonoid content. It may be challenging to distinguish between genotype-specific effects and the differences in biochemical metabolites observed between RG and the other three genotypes. For this reason, genes differentially expressed between the PL, SL, and L34 genotypes were removed from this shared gene group. This procedure, followed also by Wang et al. (2021) [11], allowed us to enrich the genes involved in the regulation of these metabolites, reducing the genes linked to specific genetic differences between genotypes. This approach allowed us to identify over a thousand DEGs (Supplementary Table S2).

The independent comparisons between genotypes revealed several OE genes related to the SG biosynthesis, as already suggested by Chen et al. (2014) [15]. In particular, *acetyl-CoA C-acetyltransferase*- and *hydroxymethylglutaryl-CoA reductase (NADPH)*-encoding enzymes are involved in the early stages of the MEP pathway (Figure 2). Interestingly, *GA2OX2*, which catalyses the 2-beta-hydroxylation of several biologically active gibberellins, was under-expressed. This could represent a biosynthetic shift from GAs to SGs [1,45]. 

DEG characterization revealed four OE genes encoding UGTs, including *UGT76G1*, known to be crucial for the SG biosynthesis [14]. Moreover, many TFs, such as WRKY6 and WRKY11, which could play a role in the regulation of SG quantitative variation [11,45,46,47], were found as over-expressed (Supplementary Table S2). Interestingly, the expression of genes within the SG and GA pathway, along with several transcription factors like MYB, bHLH, WRKY, and NAC, can impact the SG content depending on the developmental phase [45]. In addition, the expression of these TF families in *S. rebaudiana* has a crucial role in cellular morphogenesis and signalling pathways responsive to plant growth regulators [45,48].

Several genes involved in phenylpropanoid biosynthesis can undergo multiple alternative splicing modifications during biotic and abiotic stresses, and plant development [49,50,51]. In particular, high-light conditions, iron deficiency, and pathogens contribute to shaping alternative splicing patterns of genes related to the phenylpropanoid pathway [52,53,54]. PL, SL, and L34 revealed a strong over-expression of genes deputed to pre-mRNA splicing of genes encoding enzymes controlling the phenylpropanoid pathway (Figure 3). This finding suggests that these transcripts have isoforms, which may contribute to the accumulation of PCs in a pre-flowering stage or during abiotic stresses in the leaves. Furthermore, many genes encoding TFs associated with phenylpropanoid pathway regulation, such as MYB12, were over-expressed (Supplementary Table S2) [55,56,57].

The phenylpropanoid pathway may lead to structural phenylpropanoids such as lignin, lignan, suberin, and cutin [29,58]. Our gene expression data indicate that the phenylpropanoid metabolic flux was directed on lignins and lignans, as evidenced through the activation of *peroxidase*, a gene involved in the biosynthesis of monolignol formation. The over-expression of *peroxidase* could potentially lead to an enhanced production of lignin and lignans, thus more resistant leaves towards a wide range of biotic and abiotic factors, such as attacks of pathogens and mechanical stresses [59,60,61,62]. However, specific biochemical analyses should be conducted in the future to confirm this hypothesis. 

The PC content could also be influenced by plant hormones [63]. Our data go in that direction. Notably, the over-expressed *abscisic acid receptor recruitment factor* (*CAR*) gene plays a pivotal role in recruiting abscisic acid (ABA) receptors to the plasma membrane, facilitating ABA signalling. ABA triggers a specific upregulation in the transcript levels of transcription factors and genes responsible for encoding enzymes within the phenylpropanoid pathway [63]. Also, auxin plays a role in the regulation of the phenylpropanoid pathway. For example, Indole-3-butyric acid (IBA), the auxin precursor, enhances the expression of major flavonoid biosynthesis genes, such as *C4H* and *CHS*, in tea cuttings [64]. In our analyses, two genes encoding transcriptional repressors of auxin were UE, suggesting a role of this hormone in regulating PC content.

### 3.2. Co-Expression and Phylogenetic Analyses Indicate Putative Functions of UGTs

A co-expression analysis allowed the identification of a number of candidate genes involved in SGs’ and PCs’ amount regardless of the expression pattern (over- or under-expressed). The eight modules derived from the WGCNA analysis were extensively examined. However, to streamline the work, we focused on the two modules, turquoise and red, that exhibited the highest correlation with the phenotypic traits. Among them, we dedicated a more in-depth analysis on the turquoise module because it was richer in genes (449) and more informative compared to the red module (54). The turquoise module significantly correlated with Reb A, Reb E, total phenols, and total flavonoids. The core of the turquoise module is represented by 88 genes, forming a co-expression network of candidate genes, among which many have never been characterized for SG and PC biosynthesis, including a gene at the centre of the module not yet annotated (NA_Streb.Contig05310G000020.1).

The presence in the turquoise module of two *UGT76H1*, one *UGT76G1*, and one *UGT85C1*, in addition to several genes belonging to the phenylpropanoid pathway, such as *peroxidase* (*PER17*), *caffeoyl-CoA O-methyltransferase* (*CAMT*), and the *malonyl-coenzyme A:anthocyanin 3-O-glucoside-6″-O-malonyltransferase* (*3MAT*), and 21 TFs, such as *SCL3*, *WRK11*, and *MYB111*, suggests a synergistic regulatory network for the Reb A, Reb E, total phenol, and flavonoid content.

Other studies suggested the involvement of these TFs in the regulation of both SGs and PCs. In particular, MYB111 promotes the transcription of flavonoid-related genes in *Arabidopsis* [65]. Interestingly, MYBs also promote the synthesis of terpenoid compounds [66,67,68] in other species. Sun et al. (2021) [46] suggested a nitrate-MYB-terpenoid working module in nitrate-deficiency-stimulated SG biosynthesis in *S. rebaudiana.* Many studies suggest that WRKYs play a significant role in SG biosynthesis [11,45,46,47]. Moreover, WRKYs have been found to regulate the biosynthesis of phenolic compounds [30,69,70,71,72] in other species. The presence of TFs regulating auxin (*PIN3*), GA and brassinosteroid signalling (*IBH1*), and ABA (*NFXL2*) confirms an extensive gene regulatory network that includes plant hormones. Although other TFs such as *ARFC*, *BH048*, *C3H19*, *COL16*, *COL9*, *DIV*, *DSLE*, *ERF5*, *ERF82*, *PCL1*, *TGA7*, *RAP23*, and *ZHD1* were characterized for specific roles [73,74,75,76,77,78,79,80,81,82], they have not been demonstrated to regulate SG and PC biosynthesis. The potential involvement of these candidate TFs in regulating the production of these secondary metabolites deserves further investigation, for example, by editing these genes, given their role in regulating various metabolic pathways in plants and their co-expression pattern.

UGTs are involved in a number of biochemical pathways, including the glycosylation of SGs and PCs [83,84,85]. To clarify the role of UGT-encoding genes that resulted in differentially expression in our comparisons, we provided a dendrogram including UGTs with known functions taken from the literature. The dendrogram highlighted a clear separation between the UGTs involved in SG biosynthesis and PC glycosylation, clarifying the putative function of many UGTs. 

The dendrogram positioned the UGT91A1 close to UGT91D2, conferring a putative role in SG biosynthesis [16]. According to Stracke et al. (2007) [86], UGT91A1 could be involved in the glycosylation of flavonols or flavonol. Considering the promiscuous activity of UGTs towards many substrates [87], UGT91A1 may also have a function in the biosynthesis of SGs that has not yet been explored.

Sequence comparisons (Figure 6) placed UGT76H1 and UGT85C1 in the SG-related group; however, Richman et al. (2005) [14] demonstrated that UGT76H1 and UGT85C1 do not possess any catalytic activity towards steviol, steviolmonoside, steviolbioside, stevioside, or rebaudioside A. Nevertheless, we cannot exclude an active role of these UGTs in Reb E biosynthesis. The biosynthesis of Reb E, a compound with superior taste compared to stevioside, has received limited research attention. Despite its low presence in *S. rebaudiana* leaves, accounting for less than 1% of the dry mass content, Reb E offers a more pleasant flavour profile without the bitter aftertaste associated with stevioside and is approximately 150-300 times sweeter than sucrose [6,88]. To the best of our knowledge, no activity of UGT76H1 and UGT85C1 on the glycosylation of polyphenols has been reported. Thus, further molecular and biochemical investigations are needed to clarify the functional role of these two UGTs. 

## 4. Materials and Methods

### 4.1. Plant Material

For this research work, four genotypes of *S. rebaudiana* of different origins (Supplementary Table S4) and belonging to the DAFE (Department of Agriculture, Food and Environment of the University of Pisa) germplasm collection were selected from a previous study of a 3-year field trial, arranged according to a randomised block experimental design with four replications [44]. At the end of the 3rd year after planting, the leaves of the four genotypes were harvested at the pre-flowering stage (September–October 2020), when the SG content reached the highest value. For phytochemical analyses, the leaves of each sample were air-dried in a ventilated oven from 30 to 40 °C until a constant weight and the dry leaves were ground to a fine powder and stored. For the gene expression analysis, two mid-height leaves were harvested, frozen in liquid nitrogen, and stored at −80 °C.

### 4.2. Phytochemical Analyses

#### 4.2.1. Sample Extraction

Leaf powder for each sample (0.1 g) was extracted with 10 mL of 70% (*v*/*v*) EtOH and sonicated for 30 min at 60 °C. The extracts were centrifuged at the end of sonication (3500 rpm for 10 min) and filtered with a syringe filter (Ø 0.45 mm) to remove any suspended material. The extracts obtained were stored at 4 °C until subsequent analyses.

#### 4.2.2. Spectrophotometric Assays

Total phenolic content was assessed through the Folin–Ciocalteu method [89], determining the absorbance at 765 nm with a UV-Vis spectrophotometer (Varian Cary 1E, Palo Alto, CA, USA). The results were expressed as milligrams of gallic acid equivalents per gram of dry weight (mg GAE g^−1^ DW). This method measures the total concentration of phenolic hydroxyl groups in the plant extract by reducing the Folin–Ciocalteu reagent, forming a blue complex.

Total flavonoids were evaluated using the aluminium trichloride method, according to Jia et al. (1999) [90]. The flavonoids–aluminium reaction formed a pink complex measured at 510 nm using a UV-Vis spectrophotometer (Varian Cary 1E, Palo Alto, CA, USA) and the results were expressed as s mg of catechin equivalents per gram of dry weight (mg CE g^−1^ DW).

The determination of free-radical-scavenging activity (DPPH) of *S. rebaudiana* leaf extracts followed [2]. The DPPH assay is based on the reducing activity of the antioxidant molecules against the 1,1-diphenyl-2-picryl-hydrazil (DPPH) radical, characterised by a purple-red colour measured at 517 nm using a UV-Vis spectrophotometer (Varian Cary 1E, Palo Alto, CA, USA). The total free-radical-scavenging capacity was expressed as 6-hydroxy-2,5,7,8-tetramethylchroman-2-carboxylic acid (Trolox) equivalents per gram of dry weight (mmol TE g^−1^ DW).

#### 4.2.3. Steviol Glycoside Content and Composition

The extraction procedure and SG profile followed Zimmermann et al. (2012) [91]. SGs were estimated using a Jasco PU980 HPLC system (JASCO Benelux B.V., Utrecht, the Netherlands) coupled with a UV-visible wavelength detector. A hydrophilic column (Luna HILIC 200A, Phenomenex Inc., Torrance, CA, USA), 5 mm, 250 mm × 4.6 mm (Phenomenex Inc., Torrance, CA, USA), and the corresponding pre-column (4 × 3.0 mm) were used. The UV detection was performed at 205 nm at room temperature, with a flow rate of 0.68 mL/min and a run time of 20 min. Separation was carried out in acetonitrile/water (80:20) as an isocratic mobile phase at pH 3.6 regulated with acetic acid. Chromatograms were gathered online, and data were obtained using a Jasco interface (Hercules 2000 Interface Chromatography). The Common Steviol Glycosides Standards Kit was purchased from Chromadex (LGC Standards S.r.L., Milan, Italy). The calibration curves (0.005–1.00 g L^−1^) were achieved from standard mixtures containing rubusoside, dulcoside A, stevioside, and rebaudioside A, C, D, E, and M (Reb A, C, D, E, and M), and SGs were quantified using authentic standards. Total SGs were determined as the sum of single SGs [44].

#### 4.2.4. PCA Analysis

A principal component analysis (PCA) was performed on the total content of steviol glycosides, phenols, flavonoids, and DPPH activity using R. As an unsupervised method, the groups of samples obtained with PCA can be observed even when there are no reference samples that can be used as a training set to establish the model. The PCA was carried out to reduce the dimensionality of the multivariate data of the matrix whilst preserving most of the variance.

### 4.3. Transcriptomic Analyses

#### 4.3.1. RNA Extraction

Frozen leaves from three biological replicates for each of the four genotypes were used for analyses. The samples were ground in a mortar, and total RNA was isolated following the Logemann extraction protocol [92]. RNA extracted was treated with DNAse I (Roche, Rotkreuz, Switzerland) according to the manufacturer’s instructions to remove genomic DNA traces. RNA was purified with phenol/chloroform and precipitated with standard procedures. The RNA quality was assessed with an electrophoretic, spectrophotometric, and bioanalyzer analysis carried out through a Bioanalyzer 2100 (Agilent Technologies, Santa Clara, CA, USA).

#### 4.3.2. RNA Sequencing and Mapping Procedures

Total RNA was used to generate 12 RNA-Seq cDNA libraries by using the TruSeq RNA-Seq Sample Prep kit according to the manufacturer’s protocol (Illumina Inc., San Diego, CA, USA). The mRNA fraction was isolated from total RNA, exploiting the poly-A tail; then, mRNA was chemically fragmented and subsequently converted to cDNA. The libraries were sequenced with the Illumina HiSeq2000 platform (Illumina Inc.), producing 150 nt paired-end cDNA libraries. The quality of paired-end reads was checked using FastQC v0.11.5 [93], and the overall quality was refined using Trimmomatic v0.39 [94], with the following parameters: HEADCROP:19 CROP:120 SLIDINGWINDOW:4:24 MINLEN:120. Contaminating rRNA sequences were removed from all libraries by mapping against sunflower ribosomal sequences downloaded from the SILVA database [95] (https://www.arb-silva.de/; accessed on 17 February 2023). The mapping procedure was performed with CLC Genomics Workbench v9.5.3 (CLC-BIO, Aarhus, Denmark) using the following parameters: mismatch cost = 2, deletion cost = 3, insertion cost = 3, length fraction = 0.5, similarity fraction = 0.8. The reads mapped onto the sunflower rRNA were discarded. Afterwards, the high-quality 120 nt paired-end reads were mapped onto coding sequences (CDSs) of *S. rebaudiana* [43]. This high-quality chromosome-level genome represents the best choice as a reference for bioinformatic analyses in this species [96]. A mapping analysis onto CDS sequences was carried out with CLC Genomics Workbench v9.5.3 using the following parameters: mismatch cost = 2, deletion cost = 3, insertion cost = 3, length fraction = 0.9, similarity fraction = 0.9. Raw reads obtained from Illumina sequencing were deposited in the Sequence Read Archive under bioproject accession PRJNA949568.

#### 4.3.3. Differential Expression Analysis

Counted reads per transcript were converted to reads per kilobase per million reads mapped (RPKM) to calculate gene expression levels [97]. Only genes with RPKM > 1 in at least one library were considered as expressed and retained for a further analysis. The raw counts produced via mapping were analysed with the R statistical package “edgeR” in order to retrieve differentially expressed genes (DEGs) [98]. The *p*-value was corrected using the False Discovery Rate (FDR) method [99]. The fold change (FC) values were normalized using a log_2_ transformation. We considered a gene as differentially expressed (DEG) for an absolute value |log_2_ FC| ≥ 1 and an FDR value < 0.05.

#### 4.3.4. Differentially Expressed Gene Characterization

The Gene Ontology (GO) terms were obtained from the annotated *S. rebaudiana* genome (https://doi.org/10.6084/m9.figshare.14169491.v1; accessed on 17 February 2023) [43]. The GO enrichment analysis on DEGs versus the entire transcriptome was performed with Blast2GO v5.2.5 using Fisher’s exact test [100].

The KEGG Automatic Annotation Server (KAAS) was used to obtain KEGG orthologue (KO) codes for corresponding DEGs [101]. Alignment on DEG sequences was carried out using KAAS with default parameters except for the “single directional best-hit” (SBH) parameter, as described by Torre et al. (2016) [102]. Subsequently, KO id codes of corresponding DEGs were submitted to KEGG for a pathway network analysis (Kyoto Encyclopaedia of Genes and Genomes) [103]. Transcript sequences of *S. rebaudiana* were supplied to Mercator v4.3 [104] in order to detect corresponding bins for MapMan. Therefore, bins were uploaded on MapMan v3.0.0 [105] and functional classes corresponding to obtained DEGs were analysed. The heatmaps were produced using Heatmapper [106].

#### 4.3.5. Construction and Analysis of Co-Expression Networks

The R WGCNA package v1.68 was used to construct co-expression networks for DEGs [107]. Expression values (RPKM) corresponding to DEGs were used to build the co-expression network. The soft threshold power was chosen using the gradient method as suggested in the manual, allowing us to detect 16 as the best suitable value for the analysis and calculate the adjacency matrix. The eigengene dendrogram was constructed for module detection employing the dynamic tree cut method (MEDissThres = 0.1, minModuleSize = 30). A regression-based *p*-value was then used to determine the correlation between the module eigengenes and the biochemical traits to identify modules significantly correlated with previously obtained biochemical traits. The co-expression network of genes with a cutoff of 0.4 was visualised with VisANT v5.53 [108].

#### 4.3.6. Construction of UGT Dendrogram

A dendrogram based on amino acidic sequence similarity with UGTs with known functions in SG biosynthesis and PC glycosylation [47,83,84,85,109,110] was produced using MEGA X v10.1.8 [111] to characterize the *S. rebaudiana* UGTs identified in the DEGs. Sequences were aligned to each other using MUSCLE v3.8.31 [112]. The dendrogram was constructed on aligned sequences by using the Maximum Likelihood method with a bootstrap of 1000.

## 5. Conclusions

*S. rebaudiana* leaves, rich in beneficial compounds like phenolic compounds (PCs), such as flavonoids, and steviol glycosides (SGs), are gaining attention for their practical applications in nutrition and therapeutic applications. Identifying genes that synergistically promote the production of PCs and SGs is crucial for the biotechnological breeding of *S. rebaudiana*.

This work aimed to characterize four *S. rebaudiana* genotypes with phytochemical analyses and identify the genes encoding enzymes and transcription factors (TFs), putatively synergistically governing specific PC and SG biosynthesis, by using an independent pairwise comparison RNA-seq analysis of four genotypes with diverse contents of these metabolites, three with high total SG and PC content and one with a very low accumulation of such compounds. Several candidates were identified by analysing 1136 differentially expressed genes (DEGs) common to the three high-SG and -PC genotypes. UGT76G1 was confirmed as crucial for total SG content, while UGT76H1 and UGT85C1 were candidates for Reb E biosynthesis. On the other hand, the role of UGT91A1 in SG biosynthesis should be investigated.

A co-expression network analysis represents the first step to identifying the putative shared regulatory network between PCs and SGs in stevia. It showed positive correlations between DEGs and the biochemical traits of the four *S. rebaudiana* genotypes, including the most abundant SGs and total phenols and flavonoids. The core of the turquoise module comprises 88 genes, establishing a co-expression network of candidate genes. The majority of genes have not been characterized for SG and PC biosynthesis, including a gene at the centre of the module that remains unannotated (NA_Streb.Contig05310G000020.1).

The presence in the turquoise module of *UGT76G1*, *UGT76H1*, *UGT85C1*, and *UGT91A1*, in addition to several genes belonging to the phenylpropanoid pathway, such as *peroxidase* (*PER17*), *caffeoyl-CoA O-methyltransferase* (*CAMT*), and the *malonyl-coenzyme A:anthocyanin 3-O-glucoside-6″-O-malonyltransferase* (*3MAT*), and 21 TFs, such as *SCL3*, *WRK11*, and *MYB111*, suggests an extensive synergistic regulatory network implicated in the biosynthesis of PCs, SGs, and antiradical activity in *S. rebaudiana* leaves.

Although a further investigation of these genes and their interactions within this network is necessary, the genes identified in this work hold great potential for developing future breeding programmes, including gene-editing-oriented breeding of *S. rebaudiana* and other species, in order to synergistically increase the SG and PC content in the leaf.

## Figures and Tables

**Table 1:** Total SGs, total phenols, total flavonoids, and DPPH values in leaf extracts of four *S. rebaudiana* genotypes during the pre-flowering stage (September–October 2020). Values are the mean of 3 replicates ± standard error.

Genotype	Total SGs (g 100 g^−1^ DW)	Total Phenols (mg GAE g^−1^ DW)	Total Flavonoids (mg CE g^−1^ DW)	DPPH (mmol TE g ^−1^ DW)
**SL**	29.59 ± 1.74 a	118.45 ± 2.82 a	87.14 ± 4.41 b	0.749 ± 0.049 ab
**L34**	28.26 ± 2.1 a	130.20 ± 3.56 a	118.43 ± 3.66 a	0.879 ± 0.044 a
**PL**	24.85 ± 2.31 a	89.56 ± 6.58 b	91.13 ± 4.38 b	0.659 ± 0.062 b
**RG**	9.23 ± 0.79 b	41.98 ± 0.38 c	33.96 ± 2.44 c	0.196 ± 0.003 c
Significance	***	***	***	***

Values followed by identical letters are not significantly different for *p* < 0.05, according to the LSD post hoc test; The significance of variability factors according to one-way ANOVA: ***, significant at *p* ≤ 0.001 level.

**Table 2:** SG profile in leaf extracts of four *S. rebaudiana* genotypes during the pre-flowering stage (September–October 2020). Values are the mean of 3 replicates ± standard error. Values are expressed as g 100 g^−1^ DW.

Genotype	Rubusoside	Dulcoside A	Stevioside	Reb C	Reb A	Reb E	Reb M	Reb D
**SL**	1.59 ± 0.004 a	1.19 ± 0.11 b	9.07 ± 0.37 b	2.60 ± 0.15 b	12.68 ± 1.03 a	0.58 ± 0.001 b	tr	1.80 ± 0.08 b
**L34**	0.48 ± 0.03 b	2.13 ± 0.14 a	17.14 ± 1.79 a	1.58 ± 0.11 c	6.32 ± 0.25 c	0.62 ± 0.004 a	tr	nd
**PL**	0.15 ± 0.01 d	1.24 ± 0.05 b	10.24 ± 1.25 b	1.42 ± 0.06 c	9.07 ± 0.90 b	0.57 ± 0.001 c	tr	2.17 ± 0.03 a
**RG**	0.27 ± 0.001 c	2.21 ± 0.07 a	0.18 ± 0.06 c	4.76 ± 0.54 a	0.79 ± 0.07 d	nd	nd	1.01 ± 0.05 c
Significance	***	***	***	***	***	***	ns	***

Values followed by identical letters are not significantly different for *p* < 0.05, according to the LSD post hoc test; nd: not detected; tr: traces. The significance of variability factors according to one-way ANOVA: ns, not significant; ***, significant at *p* ≤ 0.001 level.

## Data Availability

RNA sequencing data have been deposited in the NCBI repository under Bioproject accession code PRJNA949568.

## Supplementary Materials

The following supporting information can be downloaded at: https://www.mdpi.com/article/10.3390/ijms25042136/s1.
