# Supplementary material for: Transcriptomic Analyses Reveal Insights into the Shared Regulatory Network of Phenolic Compounds and Steviol Glycosides in Stevia rebaudiana
Source: Int J Mol Sci. 2024 Feb 10;25(4):2136. doi: 10.3390/ijms25042136 (PMC10889303; doi:10.3390/ijms25042136)
Supplement: Supplementary file 1 [file ijms-25-02136-s001.zip › Supplementary Figure_1.pdf]

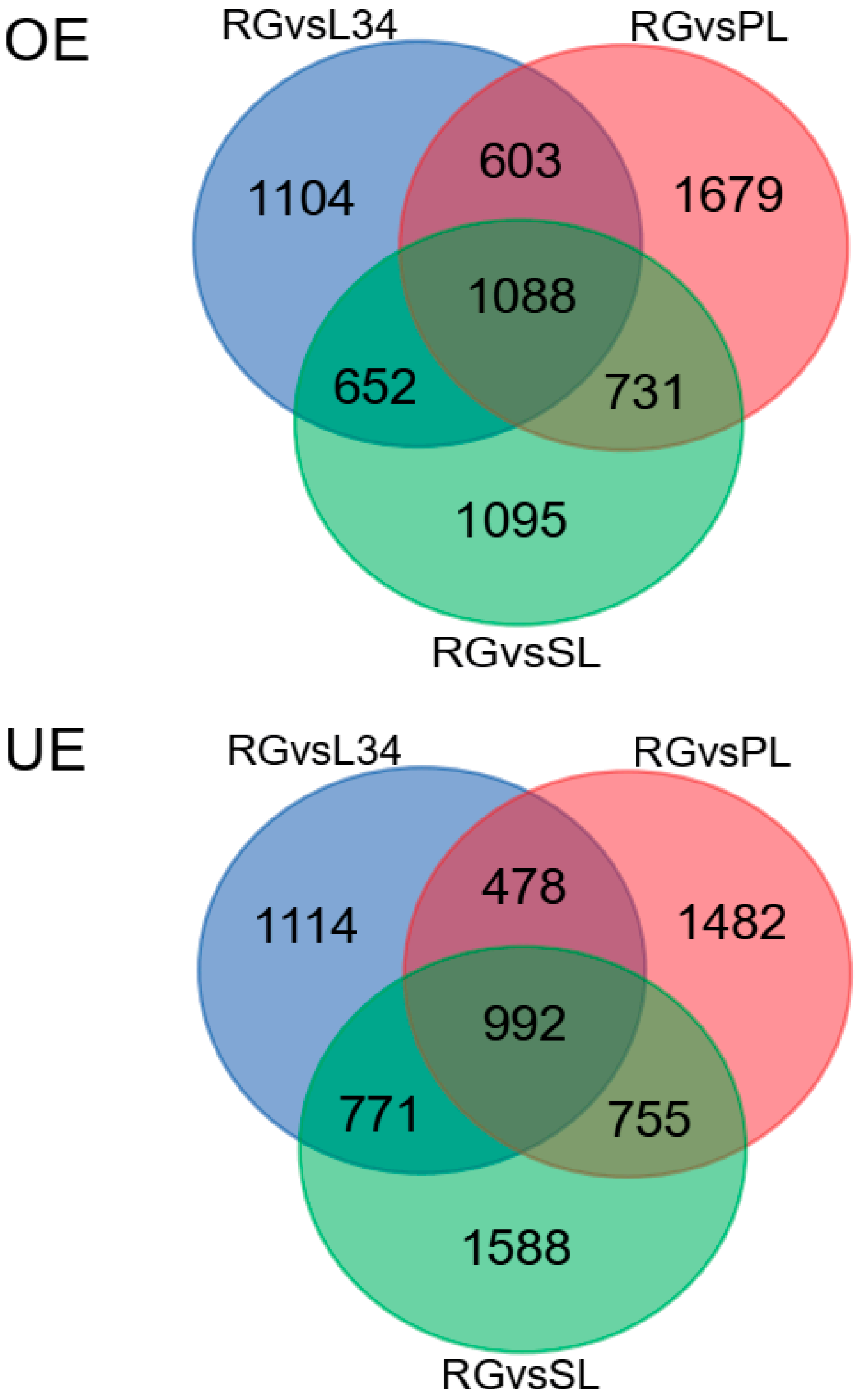

Supplementary Figure 1. Venn diagram of over (OE) and under-expressed (UE) genes of *Stevia rebaudiana* by three independent comparisons of RG to PL, SL, and L34.
