# Supplementary material for: Transcriptomic Analyses Reveal Insights into the Shared Regulatory Network of Phenolic Compounds and Steviol Glycosides in Stevia rebaudiana
Source: Int J Mol Sci. 2024 Feb 10;25(4):2136. doi: 10.3390/ijms25042136 (PMC10889303; doi:10.3390/ijms25042136)
Supplement: Supplementary file 1 [file Supplementary_Figure_5.pdf › Supplementary_Figure_5.pdf]

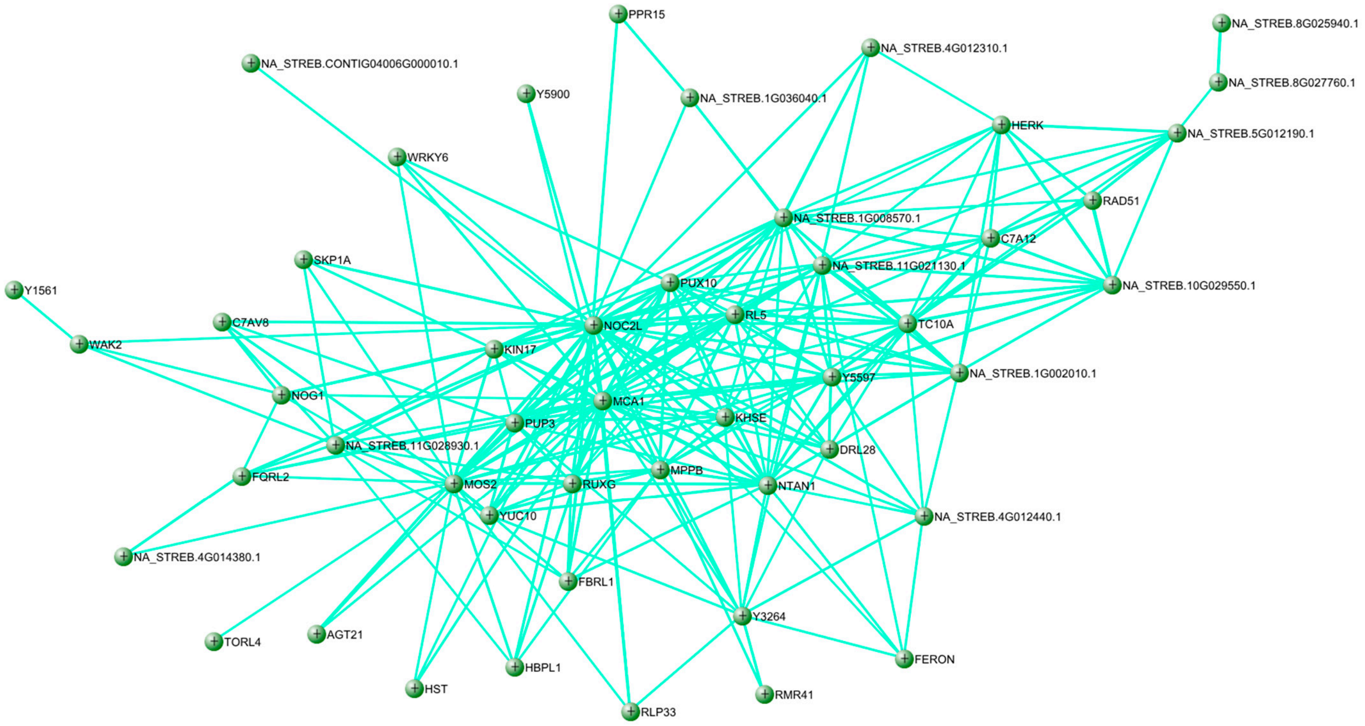

Supplementary Figure 5. Co-expression network of the red module eigengenes. The network shows the interaction that was detected between the eigengenes in the module with a cutoff of 0.15. Each node represents a gene in the network. NA: not annotated.
